# Geographic distribution of Ontario pharmacists: A focus on rural and northern communities

**DOI:** 10.1177/17151635221115411

**Published:** 2022-08-05

**Authors:** Patrick Timony, Sherilyn K. D. Houle, Alain Gauthier, Nancy M. Waite

## Abstract

**Introduction:**

Canadians living in rural and northern communities face particular health needs and challenges in accessing primary care services. Ontario pharmacists are increasingly able to optimize patient care with a broadening scope of practice; this was highlighted during the COVID-19 pandemic. This study explores the geographic distribution of pharmacists to evaluate their potential to deliver health care in rural and northern Ontario communities.

**Methods:**

A secondary analysis of the Ontario College of Pharmacists’ registry data was undertaken, with all Part A pharmacists who had at least 1 patient care practice site included in the analysis. Full-time equivalent (FTE) hours worked at each practice site were calculated and compared with the population distribution. Ratios of FTEs per 1000 residents by census subdivision (which represents communities) were calculated and compared by geography, north vs south and urban vs rural (further subdivided by metropolitan-influenced zones).

**Results:**

The greatest availability of pharmacist FTEs was found in urban communities (with slightly better availability in the north), whereas the lowest availability was found in the most rural communities. A more granular observation revealed that northern communities were more likely to have no local pharmacist access (72%) compared with southern communities (24%).

**Discussion:**

Rural and northern communities are underserved. Novel approaches to overcoming the rural pharmacist care gap include rural practice incentives, targeted enrollment of rural students, increased rural exposure in pharmacy schools and the utilization of new technologies such as telepharmacy and drone medication deliveries.

## Introduction

The health of Canadians in rural and northern communities has been a national and regional priority for decades; as a result, there is substantial research characterizing the health needs of these Canadians and the challenges they face in accessing care. Historically, research has focused on how the shortage of physicians in rural and northern communities is a key barrier to health care access.^1-3^ However, with changing scopes of practice and novel care-delivery models, it is important to also consider the availability of other health professionals, such as pharmacists.^2^ This was particularly evident during the COVID-19 pandemic.

Barriers to the provision of care in rural and remote communities include logistical challenges such as travel distance to services, weather, transportation, clinic operating hours and difficulty in booking appointments.^2,4-6^ Rural community members have also expressed cultural and interpersonal concerns regarding access to care, such as the importance of shared values and the desire for more longitudinal relationships with community health care providers; yet these same communities have higher rates of health care worker turnover, making these barriers difficult to overcome.^2^ For example, previous work has demonstrated that rural southern Ontario is distinct from rural northern Ontario with respect to physician workforce demographics, practice structures and hours worked.^7,8^

**Knowledge Into Practice**

- In Ontario, 99.4% of residents in urban communities live within 5 km of a pharmacy, compared with 40.9% of rural residents. However, little is known about the distribution and availability of pharmacists in the north compared with the south or across various degrees of rurality.
- Most northern communities (72%) have no local access to a pharmacist, compared with 24% of communities in the south. This trend is particularly pronounced in rural communities that are at a greater distance from urban centres.
- As pharmacists’ scope of practice expands, the profession is becoming increasingly relied upon to provide a number of community-based primary care services, although many rural and northern communities are underserved by pharmacists, which is a barrier to accessing these services.
- Novel approaches should be considered to overcome rural pharmacist gaps, including rural practice incentives, targeted enrollment of students with rural backgrounds, exposure to rural practice in pharmacy schools and the use of new technologies such as telepharmacy and drone medication deliveries.

Similarly, rural-urban differences in health disparities may not be uniform for all rural areas of Ontario. For example, at the provincial level, urban areas have a higher age-standardized prevalence of diabetes than rural areas do, but several areas in rural northern Ontario have a significantly higher prevalence than the provincial average, whereas areas in rural southern Ontario have a significantly lower prevalence.^9^ A similar pattern emerges when looking at age-standardized hypertension prevalence, with northern rural areas significantly above the provincial average and southern rural areas significantly below the provincial average.^9^

Given that the barriers to care and the actual health care needs of rural communities may be quite distinct from one another, even within the province, geographic analyses of health care professionals and their service delivery should be more granular than just rural versus urban if we are to better understand where and how health care capacity can be best situated.

**Mise En Pratique Des Connaissances**

- En Ontario, 99,4 % des résidents des communautés urbaines habitent à moins de 5 km d’une pharmacie, comparativement à 40,9 % pour les résidents des régions rurales. Toutefois, on ne connaît pas très bien la distribution et la disponibilité des pharmaciens dans le nord par rapport au sud ou à divers degrés de ruralité.
- La plupart des collectivités nordiques (72 %) n’ont pas accès à un pharmacien, comparativement à 24 % pour les communautés du sud. Cette tendance est particulièrement prononcée dans les communautés rurales qui se trouvent à une plus grande distance des centres urbains.
- À mesure que la portée de la pratique des pharmaciens s’élargit, la profession est de plus en plus utilisée pour fournir un certain nombre de services de soins primaires communautaires, bien que de nombreuses communautés rurales et nordiques soient mal desservies par les pharmaciens, ce qui constitue un obstacle à l’accès à ces services.
- On devrait envisager de nouvelles approches pour combler les lacunes des pharmaciens ruraux, y compris les incitations à la pratique rurale, l’inscription ciblée des étudiants ayant des antécédents ruraux, l’exposition à la pratique rurale dans les écoles de pharmacie et l’utilisation de nouvelles technologies telles que la télépharmacie et la livraison de médicaments par drone.

Ontario pharmacists are increasingly able to provide optimal patient care with a wider suite of services and certainly have demonstrated this during the COVID-19 pandemic. In fact, pharmacists, when compared with physicians, are more likely to serve patients who take medications, regardless of the size of the community in which they practice.^10^ In addition, they are available during periods of extended hours, and appointments are often not needed. It is therefore important to understand the geographic distribution of pharmacists to evaluate their potential in delivering health care to Ontarians residing in rural and northern communities. While previous work has assessed the distribution of pharmacies in Ontario by urban versus rural status,^11^ it did not consider differences between northern or southern regions and measured pharmacy presence rather than pharmacist availability to provide care. The present study characterizes the availability of pharmacists, as measured by full-time equivalent (FTE) hours worked, in communities across Ontario, including comparisons that include rural and northern considerations.

## Methods

### Data source and exclusionary criteria

Data-sharing agreements were developed between the Ontario College of Pharmacists (OCP) and the University of Waterloo, as well as between the University of Waterloo and Laurentian University, allowing confidential data transfer between OCP and the appropriate researchers. A secondary data analysis was undertaken, with OCP extracting registry data for all pharmacists licensed to provide patient care (i.e., Part A licensure). Pharmacists were excluded if 1) they had no listed practice sites, 2) they did not specify the number of hours worked at any of their listed practice sites or 3) their listed workplaces only included nonaccredited sites with no patient interaction (e.g., pharmaceutical companies, postsecondary institutions).

### Pharmacist availability

Pharmacists can practise in multiple sites across multiple communities with different hourly commitments to these sites and communities. To more accurately reflect pharmacist capacity to provide services (not just pharmacist presence at these sites), FTE hours worked at each practice site were calculated from the registry data. The OCP registry collects pharmacists’ self-reported weekly hours worked at each practice site as 1 to 14 hours, 15 to 29 hours, 30 to 39 hours and 40 hours or more. These data were converted to FTEs, assuming a 40-hour workweek, by selecting the midpoint of each range to represent the number of hours worked at each practice site. For instance, a pharmacist who reported working between 1 and 14 hours at a given practice site was estimated to have worked 7 hours at that site, which was converted to 0.175 FTEs. The same calculation was conducted for those who reported working 15 to 29 hours (22 hours estimated = 0.55 FTEs), 30 to 39 hours (35 hours estimated = 0.875 FTEs) and 40 or more hours (40 hours estimated = 1 FTE). Given the size of this data set, it is reasonable to assume that the use of the midpoint for each range, while being an overestimation for some and an underestimation for others, would provide a close approximation of pharmacist availability when averaged over the pharmacist population. The distribution of pharmacists was compared with the population distribution from the 2016 Canadian Census.^12^

### Geographic definitions

Statistics Canada’s 2018 Postal Code Conversion file (PCCF) was used to convert practice site addresses from the OCP registry to census geographies. First, the municipality, or census subdivision (CSD), within which each practice site is located was identified by cross-referencing the site’s address to the PCCF. CSDs located within the northern region of the newly identified Ontario Health Interim and Transitional Region were coded as northern, whereas all others were coded as southern. The 5 Ontario Health Interim and Transitional Regions replace the previous 14 Local Health Integrated Networks (LHINs), with geographic boundaries based on existing LHIN boundaries. As such, the previous North East and North West LHINs were combined to form the Northern Ontario Health Interim and Transitional Region.^13^

Consistent with other geographic analyses,^5,14-16^ Statistics Canada’s Statistical Area Classifications were used to define rural and urban communities. CSDs classified as census metropolitan areas (CMAs; population of 100,000 or more) and census agglomerations (CAs; population of 10,000 or more) were considered urban. All CSDs falling outside of a CMA or CA were considered rural and were further categorized by degrees of rurality using the census metropolitan-influenced zones (MIZs).^17^ The MIZ is used to describe the degree of influence a CMA or a CA has on a CSD, based on commuter flow. A CSD is a strong MIZ when 30% of its employed labour force commutes to work in a CMA or a CA, as opposed to working locally. A CSD in which between 5% and 30% of its workforce commutes is considered a moderate MIZ. In weak MIZ CSDs, less than 5% of the workforce commutes, and in no MIZ CSDs, no residents commute to work in a CMA or a CA. Although the MIZ is a measure of commuter flow, it is considered a standard measure of rurality,^18^ with weaker MIZ categories representing greater degrees of rurality. Indeed, weaker MIZ CSDs are typically located further from CMAs and CAs than stronger MIZ CSDs are.^19^ Furthermore, as it relates to the present analysis, stronger MIZ CSDs also have greater access to services offered in CMAs and CAs, such as pharmacies.

### Statistical analysis

Data in the OCP database represent the entire population of pharmacists; as such, inferential statistics were not necessary. Descriptive analyses of the distribution of all active pharmacists practising in Ontario were performed. Population size is an important consideration when comparing the availability of pharmacists by CSD, with larger populations requiring more pharmacist FTEs. As a result, ratios representing the number of FTEs offered in a community per 1000 residents were calculated, thus creating a common denominator from which to draw comparisons. Ratios such as these represent pharmacist availability (with larger ratios indicative of greater availability) and distribution (with smaller ratios representing comparatively underserved areas).

### Ethics

Research ethics approval was granted by the Laurentian University Research Ethics Board (REB file No. 6017276) and the Office of Research Ethics at the University of Waterloo (ORE No. 40518).

## Results

A total of 15,644 pharmacists were captured in the OCP registry on December 20, 2018; of these, 1998 were excluded as they were not actively practising in a patient care setting in Ontario, whereas the remaining 13,666 were retained for analysis. The overall demographics of the pharmacists indicating an active patient care practice are presented in Table 1. Women accounted for 57.9% of the pharmacist workforce. A slight majority (52.8%) were educated in Canada, 6.5% in the United States and 40.7% internationally (outside of Canada and the United States). The vast majority were practising in a community pharmacy (83.7%). A slight majority indicated practising at a single site (66.4%), so it was not uncommon for pharmacists to report splitting their time between multiple practice sites.

**Table 1:** Demographics of Part A pharmacists at patient care sites (as of December 20, 2018)

	*n*	%
**Total pharmacist population**	13,666	100
**Gender**		
Female	7904	57.9
**Years since graduation**		
Less than 1	163	1.2
1-5	1980	14.5
6-10	2138	15.6
11-20	3440	25.2
21-30	2885	21.1
31-40	2175	15.9
41-50	779	5.7
51+	106	0.8
**Country of education**		
Canada	7214	52.8
USA	888	6.5
Other	5564	40.7
Practice type*		
Community pharmacy	11,436	83.7
Hospital pharmacy	2610	19.1
Other	345	2.5
**Number of practice sites**		
1	9076	66.4
2	3066	22.4
3-5	1387	10.1
6-10	99	0.7
11-20	30	0.2
20+	8	0.1

* Multiple practice types are possible given that pharmacists can work in multiple locations and settings.

On average, CSDs in Ontario had an availability of 0.92 pharmacist FTEs per 1000 population. However, the average availability does not always reflect local realities, with many CSDs having little to no access to pharmacists. In Table 2, each CSD in Ontario is divided by geographic location (north vs south and degree of rurality) and further categorized by the availability of pharmacists, either having zero pharmacist FTEs per 1000 population (i.e., no availability), ≤0.49 FTEs/1000, 0.5 to 0.99 FTEs/1000 or ≥1 FTEs/1000. This granular observation of local realities revealed that, although the availability of pharmacist FTEs seems quite favourable in the north (0.94 FTEs per 1000 population compared with 0.92 in the south), northern CSDs are more likely to have no pharmacist access (72% of northern CSDs have access to zero pharmacist FTEs compared with 24% of southern CSDs). This corresponds to 17.6% of the northern population who have no local access to pharmacists, compared with only 1.8% of the population of southern Ontario (data not shown). The tendency for northern communities to predominantly have no pharmacist availability is observed in urban areas as well as in all degrees of rurality. Furthermore, southern CSDs were more likely to have access to 0.5 to 0.99 and >1 FTEs per 1000 population (30% and 22% of southern CSDs, respectively, compared with 9% and 17% of northern CSDs).

**Table 2:** Count of census subdivisions by geographic region and pharmacist FTEs per 1000 population

			0 FTEs/1000	≤0.49 FTEs/1000	0.5 to 0.99 FTEs/1000	≥1 FTEs/1000	Total
**NORTH**	Urban	*n*	16	3	2	8	29
		%	55	10	7	28	100
	Strong MIZ	*n*	14	1	2	2	19
		%	74	5	11	11	100
	Moderate MIZ	*n*	38	2	6	10	59
		%	68	4	11	18	100
	Weak MIZ	*n*	35	0	9	14	58
		%	60	0	16	24	100
	No MIZ	*n*	64	0	0	5	69
		%	93	0	0	7	100
	Total	*n*	167	6	19	39	231
		%	72	3	8	17	100
**SOUTH**	Urban	*n*	17	27	44	38	126
		%	13	21	35	30	100
	Strong MIZ	*n*	24	31	24	8	87
		%	28	36	28	9	100
	Moderate MIZ	*n*	18	9	17	16	60
		%	30	15	28	27	100
	Weak MIZ	*n*	5	1	2	3	10
		%	50	10	20	30	100
	No MIZ	*n*	5	0	1	0	6
		%	83	0	17	0	100
	Total	*n*	69	68	88	64	289
		%	24	24	30	22	100

FTE, full-time equivalent; MIZ, metropolitan-influenced zones.

**Figure 1:**
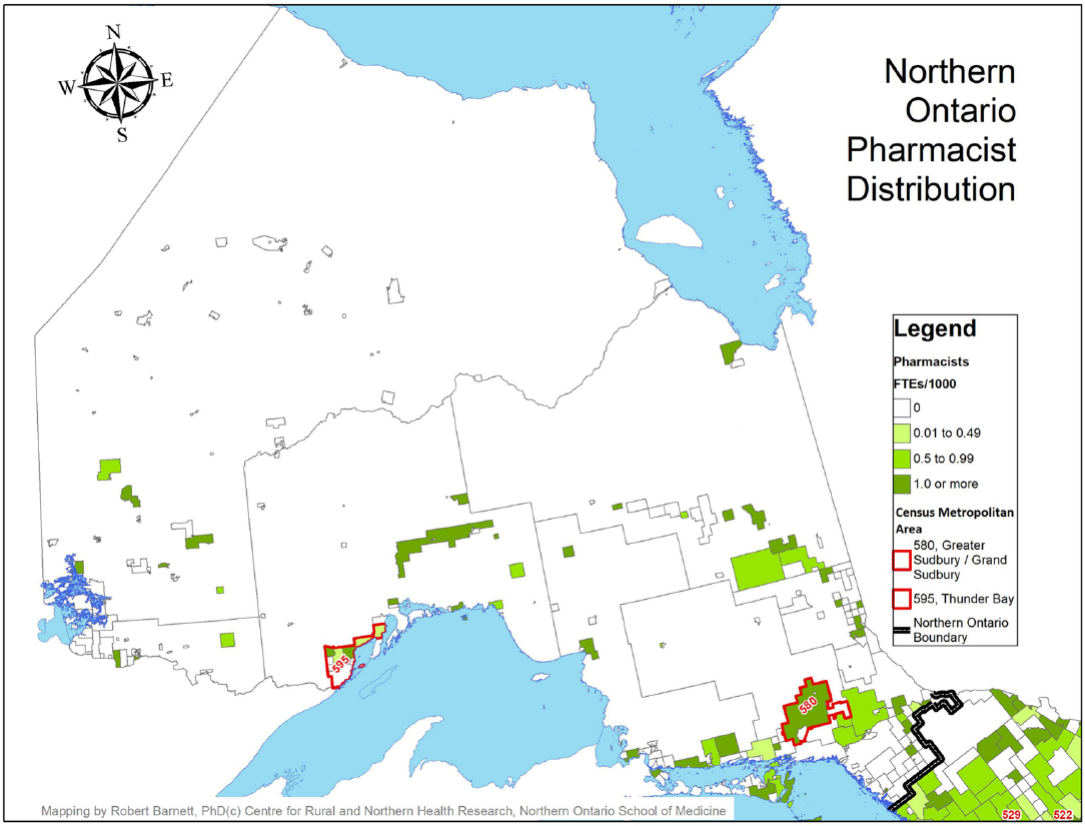
Pharmacist full-time equivalents per 1000 population, northern Ontario

**Figure 2:**
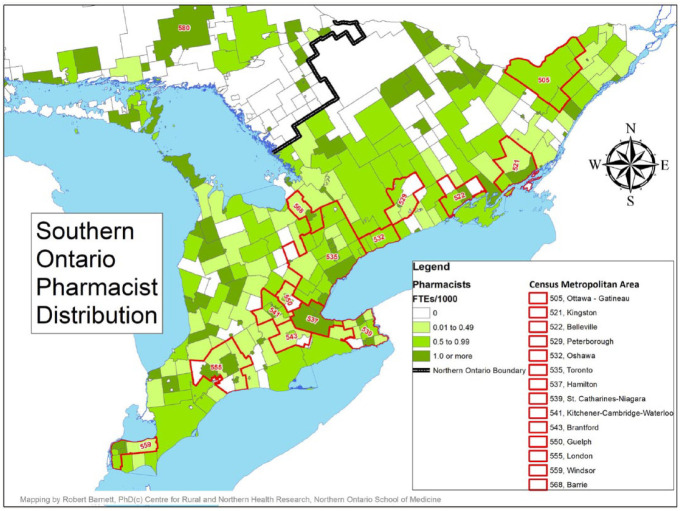
Pharmacist full-time equivalents per 1000 population, southern Ontario

A comparison of the distribution of pharmacist FTEs to the population by geographic region (north vs south and urban vs MIZ categories) is found in Table 3. Maps identifying the availability of pharmacists by CSD, with the north and south delineated with a dark black border and CMAs identified by red borders, are provided in Figures 1 and 2.

**Table 3:** Distribution of pharmacist FTEs to population by geographic region

		North		South		Total	
		Population	FTEs	Population	FTEs	Population	FTEs
Urban	*n*	496,220	517	11,446,515	10,893	11,942,735	11,406
	%	3.7	4.2	86.0	88.8	89.8	93.0
Strong MIZ	*n*	27,405	9	679,740	314	707,145	323
	%	0.2	0.1	5.1	2.5	5.3	2.6
Moderate MIZ	*n*	94,050	60	343,960	292	438,010	351
	%	0.7	0.5	2.6	2.4	3.3	2.9
Weak MIZ	*n*	119,480	123	66,915	56	186,395	179
	%	0.9	1.0	0.5	0.5	1.4	1.5
No MIZ	*n*	26,170	11	3,600	1	29,770	12
	%	0.2	0.1	0.0	0.0	0.2	0.1
Total	*n*	763,325	719	12,540,730	11,553	13,304,055	12,272
	%	5.7	5.9	94.3	94.1	100.0	100.0

FTE, full-time equivalent; MIZ, metropolitan-influenced zones.

**Figure 3:**
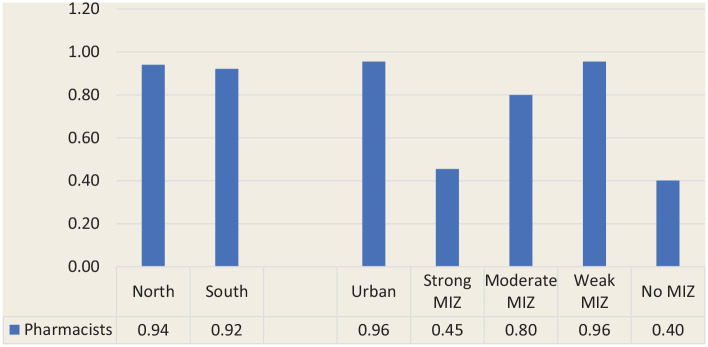
Pharmacist full-time equivalents per 1000 population by geography (north vs south) and degree of rurality MIZ, metropolitan-influenced zones.

Although the vast majority of pharmacist FTEs were offered in the southern part of the province (11,544 FTEs, 94.1%), the distribution of pharmacist FTEs compared with the population distribution was nearly identical in northern and southern regions, with 0.94 FTEs per 1000 population being offered in the north and 0.92 in the south (Figure 1). Likewise, urban CSDs had the largest availability of pharmacist FTEs (11,410 FTEs, 93.0%); however, the distribution of FTEs per 1000 population varied considerably by degree of rurality, with the most favourable ratio found in urban CSDs (0.96 FTEs per 1000 population). The availability of pharmacist FTEs drops considerably in strong MIZ CSDs (with 0.45 FTEs per 1000 population) and begins to rise as communities become more rural (with 0.80 and 0.96 FTEs per 1000 population in moderate and weak MIZ CSDs, respectively) before dropping again in no MIZ CSDs, where availability is at its lowest (with 0.40 FTEs per 1000 population; Figure 3).

**Figure 4:**
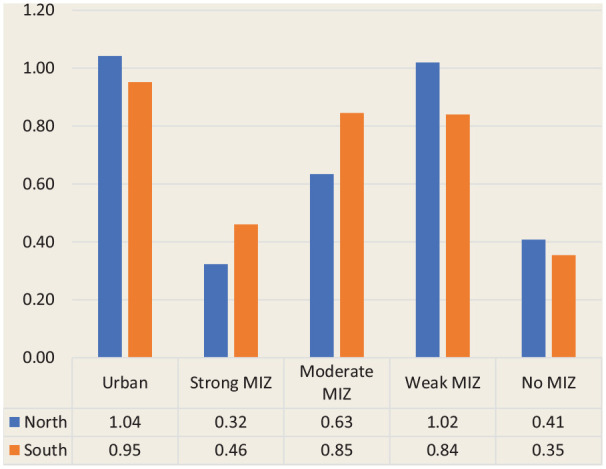
Pharmacist full-time equivalents per 1000 population by the combination of geography and degree of rurality MIZ, metropolitan-influenced zones.

The distribution of pharmacist FTEs by geography (north vs south) and degree of rurality revealed that the greatest availability was found in northern urban CSDs and northern weak MIZ CSDs (1.04 and 1.02 FTEs per 1000 population, respectively), while the lowest availability was found in northern strong MIZ and southern no MIZ CSDs (0.32 and 0.35 FTEs per 1000 population, respectively; Figure 4). However, a trend was observed both in the north and the south, whereby the availability of pharmacist FTEs is most favourable in urban CSDs, drops rapidly in strong MIZ CSDs, gradually becomes greater in moderate and weak MIZ CSDs and drops again in no MIZ CSDs.

## Discussion

This study examined the availability and distribution of pharmacists in patient care roles at a regional level in Ontario. Overall, northern and southern Ontario had similar pharmacist FTEs/population ratios, suggesting an equitable distribution of pharmacist availability between these regions. When exploring pharmacist distribution by rurality, pharmacist FTEs/population ratios were most favourable in urban areas, decreased sharply in strong MIZ communities, then gradually increased as MIZ weakens, with the exception of no MIZ communities, where pharmacist FTEs/population ratios were generally the lowest. Similar trends were observed in the north as well as the south. Although the sudden drop of pharmacist availability in strong MIZ communities seems alarming, the distribution of pharmacists by degrees of rurality tends to correspond to commuter patterns. In strong MIZ communities, a large portion of the workforce commutes to work in an urban core, where they can access pharmacies and other health care services. As a result, there is a reduced need for pharmacist services in strong MIZ communities. As fewer residents commute, access to urban-based pharmacies becomes more limited and local access becomes more essential, hence the increases in pharmacist availability observed in moderate and weak MIZ communities. However, the most rural communities continue to be the most underserved, with no MIZ communities having no commuter access to urban pharmacists while simultaneously experiencing the most unfavourable access to local pharmacists, with the smallest pharmacist FTEs/population ratios.

Although at a macro level, the distribution of pharmacists appears rather equitable between the northern and southern regions of the province, a more granular observation reveals important distinctions. When exploring pharmacist access at the community level, northern communities were far more likely than southern communities to have no local access (74% vs 24% had 0 FTEs/1000 population), with similar disparities observed at all degrees of rurality. Overall, the present study concludes that rural communities are comparatively underserved, both in terms of experiencing less favourable pharmacists FTE to population ratios and in terms of being more likely to have no local pharmacist access. However, rural access gaps are experienced differently in the north than in the south. While the south had less favourable pharmacists FTE to population ratios (particularly in weak and no MIZ communities), northern communities were far more likely to experience an absence of local access. Consequently, southern rural residents are more likely to have some local pharmacist access; however, this access may be limited in availability (i.e., fewer FTEs/population being offered), thus requiring travel to a neighbouring community to meet urgent needs. In contrast, northern rural residents are more likely to have no local pharmacist access, and if pharmacists are present within a rural community (particularly in weak and no MIZ communities), they tend to be more available (i.e., offer more FTEs/population).

Although a rural access gap is certainly present in the southern region of the province, it could be argued that this gap is more problematic in the north. For instance, a greater proportion of the northern population has no local pharmacist access and must leave their home communities to access pharmacist services. Accessing such northern neighbouring services can be particularly problematic. First, the north has more than 2.5 times as many communities classified as moderate, weak or no MIZ as compared with the south, whereas the south has more than 4.5 times as many strong MIZ communities as compared with the north, indicating that commuting is far more common in the south and that urban-based pharmacies are more accessible. The absence of pharmacists in many northern communities means residents must travel to access care and make additional trips that may not be part of their regular routine. Greater distances between communities, an absence of public transportation options and challenging driving conditions (e.g., fewer major highways and poor weather and road conditions) further limit northern access to neighbouring and urban pharmacist services. Second, the distribution of pharmacists reported in this study closely resembles that of primary care physicians, who are also predominantly located in southern and urban regions of the province.^7^ However, it is not known whether the communities that are underserved by physicians are also underserved by pharmacists. Future research in this field should combine and explore the distribution of multiple primary care providers (e.g., physicians, pharmacists, nurse practitioners) for a more nuanced understanding of primary care access in rural areas and to identify primary care deserts. Similar urban/rural health professional distributions have been observed among other Canadian jurisdictions^20,21^ and in other developed countries.^22^ In 2017, a Rural Road Map for Action was developed to provide a “guiding framework for a pan-Canadian approach to physician rural work force planning as well as improved access to rural health care,” including a goal of interprofessional care.^23^

The gap in primary care observed in rural and northern Ontario could be further compounded by the growing pharmacist scope of practice. As the role of the pharmacist has expanded to include medication management activities (e.g., initiating or adapting prescriptions), management of chronic diseases, vaccine administration and the ability to order and interpret laboratory tests related to monitoring medication outcomes,^24^ underserved communities are therefore able to access fewer primary care services than neighbouring communities are. As pharmacists have increasing primary care and public health roles, limits in their availability in a region may also contribute to furthering rural health outcome gaps. However, this growing scope of practice also provides pharmacists with the tools needed to provide optimal primary care. Pharmacists have been particularly visible during and before the pandemic due to their high degree of accessibility, being declared essential workers; the closure or shift of other primary care provider practices to virtual care; and an expanded scope of practice related to COVID-19 testing and vaccine administration. There is plenty of evidence to show the value that pharmacists can add to patient care, particularly in rural and underserved areas. For instance, rural pharmacists have been shown to improve vaccine accessibility,^25^ hypertension management^26^ and access to hormonal contraception.^27^ Furthermore, a pharmacist prescribing for an ambulatory conditions or minor ailments program in Saskatchewan was associated with cost savings for the health care system as well as improved access for patients,^28^ with a similar program currently under development for Ontario.^29^

Numerous strategies can be used to help alleviate gaps in rural and northern pharmacist access. First, practice in rural and underserved regions can be incentivized. For example, in an effort to help curb pharmacist shortages, Australia has implemented rural incentive programs that include continuing professional education allowances, which provide financial support to allow rural pharmacists to attend professional development activities; emergency locum services, which provide rural pharmacists with locum support; and intern incentive allowances, which allow rural pharmacies to retain pharmacists beyond their initial intern period.^30^ Second, targeted recruitment, retention and education strategies have been found to improve the rural supply of primary care providers. A scoping review from Australia revealed that strategies such as establishing pharmacy schools in rural areas, exposing learners to rural content in the curriculum, providing rural practice experiences during placements and enrolling students from rural and underserved communities have been established to helped increase the rural pharmacist workforce.^31^ Similar approaches have been implemented with success in Ontario to improve the supply of rural and northern physicians. Namely, the Northern Ontario School of Medicine enrolls learners with rural and northern backgrounds and provides experiential learning opportunities in rural and northern areas.^32^ This has been found to improve the likelihood of future rural and northern practice.^33^ Third, since it is unrealistic to expect pharmacists to be equally distributed and accessible in every community throughout the province, modern technologies may provide alternative opportunities to reach Ontario’s most underserved communities. For instance, telepharmacy (i.e., the use of telecommunication technologies to provide pharmaceutical services) allows pharmacists to provide clinical interventions and education from a distance and has been linked to improvements in chronic disease management and patient self-management^34^ as well as reductions in medication errors.^35^ Furthermore, telepharmacies have been found to be equivalent in quality to traditional pharmacies in terms of impact on medication adherence and appropriateness.^36^ Finally, recent innovative research is exploring the use of unmanned aerial vehicles (i.e., drones) for use in medication deliveries.^37^ Drones are considered a cost-effective alternative to the delivery of life-saving medication in areas where traditional road deliveries may be limited. A combination of telepharmacy and drone delivery may allow pharmacists to provide high-quality care to underserved communities without the need for additional travel or the establishment of a local pharmacy.

### Limitations

The use of OCP data may be considered a limitation of the present study, as it is self-reported by pharmacists, does not provide a log of actual hours worked and is represented by categorical ranges of hours. Ratios at the population level are reasonable, as pharmacists tend to offer services to an entire geographic region (not only to rostered patients, as some physicians do); however, we cannot account for other factors that may limit local pharmacist access, such as operating hours and patient access to public transportation, which vary considerably, particularly between rural and urban areas.

## Conclusion

With a growing scope of practice, and as the COVID-19 pandemic has demonstrated, Ontario pharmacists will continue to relieve pressures on the health care system, improve access to community-based primary care and, ultimately, improve health outcomes of individuals, but only if patients can access their services. At first glance, the general distribution of pharmacists by geographic location appears equitable. However, a closer look reveals several gaps in pharmacist coverage. Of note, the most rural and remote communities, with no ability to quickly access urban-based serves (i.e., no MIZ communities) are the least well served. Such communities are far more common in the North. In addition, communities with no pharmacists at all are far more prevalent in northern Ontario than in the south. In conclusion, persons residing in isolation in Ontario often face greater health challenges, and according to the results of this study, their health issues may be further compounded by the limited access to support services provided by pharmacists. ■
